# Supplementary material for: Epidemiological distribution of Echinococcus granulosus s.l. infection in human and domestic animal hosts in European Mediterranean and Balkan countries: A systematic review
Source: PLoS Negl Trop Dis. 2020 Aug 10;14(8):e0008519. doi: 10.1371/journal.pntd.0008519 (PMC7440662; doi:10.1371/journal.pntd.0008519)
Supplement: S1 Text — (DOCX) [file pntd.0008519.s002.docx]

**Search strategy**

**MEDLINE (PubMed) (1966 to 19 October 2019)**

((((((((("Echinococcosis"[Title/Abstract] OR "Hydatidosis"[Title/Abstract] OR "Hydatid Cysts"[Title/Abstract]))) OR (“Echinococcus”[Title/Abstract] OR "Echinococcus granulosus"[Title/Abstract] OR ”Echinococcosis”[Title/Abstract])) OR "Echinococcosis, "[Mesh]) OR (("Echinococcus"[Mesh]) OR "Echinococcus granulosus"[Mesh]))) AND ((((("Cross-Sectional Studies"[Mesh]) OR "Prevalence"[Mesh] OR “Incidence”[Mesh]) OR "Epidemiologic Studies"[Mesh])) OR (“prevalence”[Title/Abstract] OR "cross sectional studies"[Title/Abstract] OR "cross-sectional stud*"[Title/Abstract] OR “incidence”[Title/Abstract] OR "epidemiologic studies"[Title/Abstract] OR "epidemiologic stud*"[Title/Abstract])))) AND (("Europe"[Mesh]) OR “Europe”[Title/Abstract])

**Embase (1974 to 19 October 2019)**

#1 'echinococcus'/exp OR 'echinococcus granulosus'/exp OR 'hydatid cyst'/exp

#2 'echinococcosis' OR 'hydatidosis' OR 'hydatid cysts ' OR echinococcus OR 'echinococcus granulosus':ti,ab

#3 #1 OR #2

#4 'prevalence'/exp

#5 'prevalence' NEAR/5 study

#6 #4 OR #5

#7 #3 AND #6 AND [embase]/lim
