# Supplementary material for: Epidemiological distribution of Echinococcus granulosus s.l. infection in human and domestic animal hosts in European Mediterranean and Balkan countries: A systematic review
Source: PLoS Negl Trop Dis. 2020 Aug 10;14(8):e0008519. doi: 10.1371/journal.pntd.0008519 (PMC7440662; doi:10.1371/journal.pntd.0008519)
Supplement: Attachment — Submitted filename: Responses to Reviewers_12.06.2020.docx [file pntd.0008519.s006.docx]

Padova, 12^th^ July 2020

**Responses to Reviewers' comments**

Dear Editor,

We are grateful to reviewers for their detailed revision of our manuscript and for the useful comments on how to improve it. Please receive attached the revised version of the manuscript and here below the answers to the points raised by reviewers.

On behalf of all co-authors, I send you our best regards,

Rudi Cassini

**Methods**

Reviewer #1: The objective of the study are clearly defined, the study design is appropriate, the sample size (papers for a systematic review) sufficient to address the hypothesis being tested, and the statistical analysis is correct. There are no concerns on ethical and regulatory requirements.

Reviewer #2: Objectives are clearly articulated. Study design is appropriate and statistics are correct for the analysis performed

Reviewer #3: The first sentence of the Materials and Methods refers to “the research question”. However, a research question is not explicitly stated.

**Reply: We have now added in the first paragraph of the “Search strategy” section of the Materials and Methods a first sentence defining clearly the research question (Lines 105-107)**

Please indicate why the specific databases (and web search engines) were chosen and why the original search dates differed between sites. It is also not clear what #20-#24 refer to in the Embase search strategy (S1 text).

**Reply: Databases and web search engines were defined according to the AMSTAR checklist to assess the quality of systematic review (**[**https://amstar.ca/Amstar_Checklist.php**](https://amstar.ca/Amstar_Checklist.php)**), which indicates (#3): *“Was a comprehensive literature search performed? At least two electronic sources should be searched. The report must include years and databases used (e.g., Central, EMBASE, and MEDLINE). Key words and/or MESH terms must be stated and where feasible the search strategy should be provided. All searches should be supplemented by consulting current contents, reviews, textbooks, specialized registers, or experts in the particular field of study, and by reviewing the references in the studies found. Note: If at least 2 sources + one supplementary strategy used, select “yes” (Cochrane register/Central counts as 2 sources; a grey literature search counts as supplementary)”.* Both EMBASE and MEDLINE were searched on October 2019, which is the end-date of the search, as indicated, and the search started from the time of the first record of each database; they differ in the initial date of the search because the two databases store literature from different dates, 1966 for MEDLINE and 1974 for EMBASE. Concerning specifically the web search engines, those considered most widely covering the target publications were chosen.**

**Regarding the Embase strategy, we thank the reviewer for spotting the mistake, as “#20-#24” was a remaining fragment from a previous version. We have now included the correct version.**

Please elaborate on why only the first 500 search results were considered for the Google Scholar, Scopus, and Open Grey searches? Where these sorted by relevance or date?

**Reply: The web search engines results were sorted by date. The arbitrary number or the first 500 search results was chosen as it reasonably included all relevant results for the investigated time frame. This information has been now added to the text for clarity (Lines 114-116).**

What is meant by “publication status” (line 111)?

**Reply: published or just accepted for publication. This information has been now added to the text for clarity (Line 119).**

It appears that data could be included from 1980 onward, but only articles published after 2000 were included in the review. How do the authors justify the 1980 data cutoff (line 115)?

**Reply: Papers published from year 2000 onward may include data referring to previous years. In two occasions, 2 papers were published after year 2000 but they analysed data referring to years before 1980. We decided to exclude these two papers as the data would not fit with the timeframe investigated in our study. Now, for clarity, the range of years of the data covered by the included papers has been indicated in the tables.**

The authors need to provide an overview of the NUTS system and elaborate on what constitutes the NUTS1 and NUTS2 levels. Without this information in the main text, the paper becomes very difficult to follow.

**Reply: A brief overview of the NUTS system has been inserted in the revised paper at Lines 167-171 and the pre-existing text slightly modified. In doing this, we discovered a mistake in the number of NUTS1 composing the study area, which is 46 and not 56. This figure has been now corrected in the revised manuscript (Line 171).**

I would suggest that the NUTS1 codes be included in the actual paper (or the NUTS1 codes swapped for the region names in the tables).

**Reply: we agree with the reviewer’s suggestion to report NUTS1 codes alongside with the names of different territories cited in the text. On the contrary, we prefer not to insert in the tables the region names. Official names of the NUTS system are often not really explanatory and their excessive length will make both tables and figures poorly understandable.**

--------------------

**Results**

Reviewer #1: The analysis presented match the analysis plan. The results are clearly presented including tables and maps of good quality for clarity.

Reviewer #2: Results are clearly and completely presented. Figures and tables are clear and well presented

Reviewer #3: Since data appear to be included from 1980 onward, it would be helpful if the tables provided the time frames encompassed by the included studies.

**Reply: In many cases, each line of the table reports data from several studies, making more complicated to show clearly the timeframe of extracted data. However, we agree with the reviewer that this information is important and not explicitly found throughout the whole manuscript at present (it can be only found in the supplementary information). Therefore, we added a new column to each table, titled “years”, reporting the data requested. Footnotes are also integrated to specify that the time frame refers to the whole period covered by the studies, from the first year of the oldest one to the last year of the most recent.**

It appears that, in many publications, disease frequency (e.g., incidence) was calculated by the original authors. Did the review authors use any method to assess level of bias in the included articles?

**Reply: We did not perform a formal quality and bias assessment of publications included in the review. This limitation is now clearly indicated at the end of the first paragraph of the Discussion (Lines 350-353).**

Figure 5- I assume the authors mean E. granulosus s.l. prevalence and not CE prevalence since dogs are infected with the adult form of the parasite.

**Reply: We thank the reviewer for noting this mistake. The term CE has been changed to *E. granulosus* s.l. in the Fig 5 and in its caption, and also in other parts of the text where we referred to prevalence of the infection in general.**

Figures- prevalence and incidence bins are not mutually exclusive (e.g., would an area where the prevalence in cattle is 4.0% go in the 0.0-4.0 bin or the 4.0-10.0 bin).

**Reply: All figures have been modified accordingly.**

--------------------

**Conclusions**

Reviewer #1: The conclusions are supported by the data presented. However, the limitation of the analysis is not clearly described. The topic is of public health importance and the public health relevance is well addressed.

Reviewer #2: See summary and general comments

Reviewer #3: The authors discuss how differences in the clinical presentation of CE patients (e.g., surgical patients versus patients being treated medically or detected incidentally) impact the available data. That being said, it would be helpful if data type was included in table 1. Along those same lines, inclusion of diagnostic technique would assist with understanding the values produced for definitive hosts (table 5)?

**Reply: We agree with the reviewer that this information can improve the level of completeness of data presented in the mentioned tables. However, tables are organised aggregating data at NUTS1 level. This means that more studies, possibly with different characteristics in terms of performed diagnostic technique, can share the same row. To our opinion it is difficult to present these aspects in a clear way in the tables, maintaining an acceptable readability. All requested data are available in the supplementary material (S2 and S3), and we believe that this represents a good compromise.**

Again, please clarify the time frame for the data included in the review (line 432).

**Reply: Now we inserted the exact time frame, referring to extracted data (Line 458).**

The authors spend quite a bit of the Discussion trying to explain relationships that they earlier state could not be evaluated (e.g., in support of the authors’ previously published hypothesis regarding the use of cattle as sentinel species). Therefore, they may want to temper the language somewhat.

**Reply: During the preparation of the manuscript we discussed among ourselves on the opportunity to extract data from the tables to support the hypothesis that CE prevalence in cattle and human incidence could correlate quite well. The indication of numerical ranges appeared to us as the first step towards a more robust analysis of the relation between these two parameters, to be examined in depth in future studies. However, we were aware that this part of the discussion was a bit speculative and based on a limited sample and on descriptive data. The reviewer’s comment induced us to reconsider our decision and therefore the part of the discussion reporting the correspondence between bovine prevalence and human incidence ranges has been deleted in the revised version. The hypothesis is still present and supported by visual interpretation of figures and tables data, but it actually needs more robust analysis for providing the readership with appropriate figures to describe the relation between the two parameters.**

--------------------

**Editorial and Data Presentation Modifications**

Reviewer #1: Minor revision

Reviewer #2: (No Response)

Reviewer #3: Title: It appears that the authors not only evaluated cystic echinococcosis (i.e., infection with the larval form of the parasite), but also adult parasite infection in definitive hosts. This is not reflected in the title.

**Reply: We agree also in this point with the reviewer’s comment on the appropriateness of the used terminology. Title has been modified referring to the parasite and not to the disease caused by the larval stage.**

Abstract: NUTS1, NUTS2, and epidemiological unit are not well defined in the abstract. A clearer link needs to be made between the presented principal findings and the conclusion that Italy, Spain, and Eastern Europe are the most affected areas.

**Reply: An improvement in the presentation of the aspects mentioned by the reviewer has been attempted, taking into account the limited number of words to be used in the abstract.**

--------------------

Summary and General Comments

Reviewer #1: The manuscript PNTD-D-20-00593 by Tamarozzi et al. titled “Epidemiological distribution of human and animal cystic echinococcosis in European Mediterranean and Balkan coutries: a Systematic Rewier” describes the results obtained from a systematic literature review on Echinococcus granulosus in humans and animals. The aim of this study is to give an overview on the distribution and epidemiology of cystic echinococcosis in Europe, and specifically in European Mediterranean and Balkan countries, where this disease is endemic. For this purpose, the authors considered all possible studies that could help to answer the research question. Thus, cross-sectional and longitudinal studies, as well as case series, reporting prevalence or incidence of human or animal (ovine, bovine, and canine) E. granulosus s.l. infection were included. To ensure maximum data accuracy, only papers/studies reporting the species of Echinococcus investigated, the number of people/animals

surveyed, the number of positive cases, details about the methodology of diagnosis and the geographical site were included. Each data obtained, separated for host species, was then referred to a specific NUTS (Nomenclature of Territorial Units for Statistics). The work is presented according to the recommendations of the Preferred Reporting Items for Systematic Reviews and Meta-Analyses.

The topic is of interest due to the impact of cystic echinococcosis (CE) on human and animal health, but also for the economic losses associated. There is a growing interest on CE and a comprehensive review of its distribution in definitive and intermediate hosts in endemic areas, as those described in the work, could improve the targeting of control measures and to enhance the cost-effectiveness of integrated disease control programmes for E. granulosus.

Thus, I recommend the publication of the paper in PLOS Neglected Tropical Diseases after minor revisions. The paper is well written, the questions posed by the authors are well defined, the methods appropriate, and the results, discussion and conclusions well balanced and adequately supported by the data.

**Reply: We thank the Reviewer for appreciating the relevance of the topic and our work**

GENERAL COMMENTS

-	Authors should explain why they didn’t consider animal intermediate hosts other than cattle and sheep, e.g. swine and equids. Furthermore, the role of sylvatic canids and wild intermediate hosts (e.g. wild boar and wild ungulates) should be considered in the epidemiology of E. granulosus.

**Reply: Both these aspects are now discussed at Lines 492-499, recognising that the exclusion of other domestic and wild species may represent a limitation of the study. However, considering the complexity of the systematic review approach, we decided to concentrate on animal hosts with recognised primary epidemiological roles, which in turn was reflected in a wider and more abundant available literature.**

-	I was wondering how the authors have treated farm prevalence data in their analysis.

**Reply: Studies reporting bovine and ovine CE prevalence rates only at farm level were excluded from the analysis, given the impossibility of using these data in the calculation of the pooled prevalence.**

-	I would suggest the authors to add data on genotypes of E. granulosus if available from the studies used for their review.

**Reply: A quantitative description of the distribution of the different genotypes of *E. granulosus s.l.* could be very interesting, mostly in the human population. In our review, 8 papers on animal intermediate hosts (genotypes G1, G2, G3 and G5 were found) and 3 articles on the definitive host (only G1) reported data on genotypes, but none of the studies on human infection, eligible to be included in the review, reported data on genotypes. The limited novelty of our findings and the paucity of relevant data on this aspect prevented us from developing more the genotype issue in the discussion. This is now presented in a more detailed way at Lines 488-491.**

-	The Authors often mention the limitation of the studies used in their review. But what about the limitation of their approach?

**Reply: We agree with the Reviewer that a clearer indication of the limits of the study itself, besides those deriving from the material available, is important. We have now amended the text including the limitations deriving from the fact that we did not perform a formal quality and bias assessment of the papers included (Lines 350-353), and that we did not explore the epidemiology of CE in livestock other than sheep and cattle that can have a role in domestic cycles and therefore human exposure to infection (Lines 492-495). We have also mentioned and explained clearly the absence of investigation of epidemiology in wild animals (Lines 496-499).**

-	From a practical point of view, I would suggest to emphasize how the maps and the outcome of the review will be transferred to policy makers and stake holders, in order to improve surveillance of this important neglected disease as well as in order to plan effective control measures against E. granulosus

**Reply: We have inserted in the Discussion (Lines 349-350) and conclusions (Lines 528-530) a brief mention to the practical use that may derive from our results.**

SPECIFIC COMMENTS

- Line 94: At the end of the introduction, the authors explain the aim of this work, specifying that the systematic review was carried out between 1980 and 2019, whereas in the abstract they mention “between 2000 and 2019”. Moreover, since papers before 2000 were not considered in the review, I would suggest to change to “between 2000 and 2019” throughout the study.

**Reply: We have amended the text for clarity, according to the suggestion of the Reviewer**

- Line 107: Please change “Echinococus” to “Echinococcus”.

**Reply: Amended.**

- Line 117, Please provide a reference for PRISMA

**Reply: We have included the reference to the PRISMA initiative website.**

Reviewer #2: In general, this is a very interesting review on the status of CE in human and animla hosts, and gives a good idea on the underrreporting of official data. The highlights of this systematic review (underreporting, centinel animals and geograpical distribution) are of importance not only for the scientific community, but also for the policy makers.

Minor comments: Authors should doublé-check if articles on animal CE refer to animals only slaughtered in a given geographical area or breeded/fed in the geographical area or reference for each article. Slaughterhouses in EU are now big and are usually "specialized" in a specific animal species, and usually receive animals of that species from all geographical regions inside the country. Geographical origin (and not place of slaughter) of the CE infected animals should be cross-checked.

**Reply: In most papers the geographical origin of the slaughtered animals was explicitly reported. However, in four studies only the slaughterhouse location was clearly indicated. In three of these papers, the text of the article allowed us to identify with a good level of certainty the NUTS1 area of provenance of slaughtered animals. In the fourth one, data were referred to the national level.**

A second question of concern is the age of animals. Sheep reaching slaughetrhouses and subjected to inspection are in many cases very young animals. Maybe this could explain the best matching between human-cow numbers and not between human-sheep numbers. This should be commented in the text

**Reply: This aspect is now better discussed at the Lines 480-482.**

Reviewer #3: This is a systematic review looking at Echinococcus granulosus senu lato infection in humans and animal hosts in Mediterranean and Balkan countries. The authors use the Nomenclature of Territorial Units for Statistics (NUTS) to analyze and present their findings. However, the NUTS system (and the reason for using this system) is not well described, making it difficult for the reader to interpret what the findings mean from a country level and control perspective.

**Reply: In the revised manuscript we improved the description of the NUTS system and the reason for using it, following previous comments from the reviewer. We believe that now the clarity in the presentation of our findings has been improved.**
