# Supplementary material for: Epidemiological distribution of Echinococcus granulosus s.l. infection in human and domestic animal hosts in European Mediterranean and Balkan countries: A systematic review
Source: PLoS Negl Trop Dis. 2020 Aug 10;14(8):e0008519. doi: 10.1371/journal.pntd.0008519 (PMC7440662; doi:10.1371/journal.pntd.0008519)
Supplement: Attachment — Submitted filename: Responses to Reviewers_22.06.2020.docx [file pntd.0008519.s007.docx]

Padova, 22^nd^ June 2020

**Responses to Reviewers' comments**

Dear Editors,

You can find here below the answer to the point raised in your last communication.

Best regards,

Rudi Cassini

**Methods**

There is a minor suggestion. For human incidence, authors should always use "annual incidence per 100,000" rather than "incidence per 100,000". This is to standardize disease reporting and to ensure the incidence is reported as annual incidence and not over some other period of time.

**Reply: We have now amended Table 1, Figure 2, abstract and the main text of the manuscript inserting the word “annual” whenever necessary, as per your suggestion.**
